# Computational neuroscience across the lifespan: Promises and pitfalls

**DOI:** 10.1016/j.dcn.2017.09.008

**Published:** 2017-10-13

**Authors:** Wouter van den Bos, Rasmus Bruckner, Matthew R. Nassar, Rui Mata, Ben Eppinger

**Affiliations:** a Center for Adaptive Rationality, Max Planck Institute for Human Development, Berlin, Germany; b Department of Psychology, University of Amsterdam, Amsterdam, The Netherlands; c Department of Education and Psychology, Freie Universität Berlin, Berlin, Germany; d International Max Planck Research School LIFE, Berlin, Germany; e Department of Cognitive, Linguistic, and Psychological Sciences, Brown University, Providence, USA; f Center for Cognitive and Decision Sciences, Department of Psychology, University of Basel, Basel, Switzerland; g Department of Psychology, Concordia University, Montreal, Canada; h Department of Psychology, TU Dresden, Dresden, Germany

**Keywords:** Computational neuroscience, Reinforcement learning, Risk-taking, Decision-making, Brain development, Identification, Strategies

## Abstract

In recent years, the application of computational modeling in studies on age-related changes in decision making and learning has gained in popularity. One advantage of computational models is that they provide access to latent variables that cannot be directly observed from behavior. In combination with experimental manipulations, these latent variables can help to test hypotheses about age-related changes in behavioral and neurobiological measures at a level of specificity that is not achievable with descriptive analysis approaches alone. This level of specificity can in turn be beneficial to establish the identity of the corresponding behavioral and neurobiological mechanisms. In this paper, we will illustrate applications of computational methods using examples of lifespan research on risk taking, strategy selection and reinforcement learning. We will elaborate on problems that can occur when computational neuroscience methods are applied to data of different age groups. Finally, we will discuss potential targets for future applications and outline general shortcomings of computational neuroscience methods for research on human lifespan development.

## 1. Introduction

Over the past two decades there has been a significant increase in the number of cognitive neuroscience studies of lifespan development (Amso and Scerif, 2015; Li and Rieckmann, 2014; Samanez-Larkin and Knutson, 2015). However, despite this inflation in empirical studies, there has been a serious lag in the development of comprehensive theories linking brain development and behavior (Pfeifer and Allen, 2012, 2016; van den Bos and Eppinger, 2016). The current conundrum of developmental neuroscience consists of two major explanatory problems. First, there is a specificity problem: The current verbal theories of neurocognitive development are not specific enough to be translated into precise behavioral and neuroscience predictions. As a result, it is often impossible to tell whether new neuroscientific data confirm or falsify existing theories (van den Bos and Eppinger, 2016). Related to this issue is the identity problem: We are often unable to precisely identify the processes that underlie developmental differences in behavior.

**Fig. 1:**
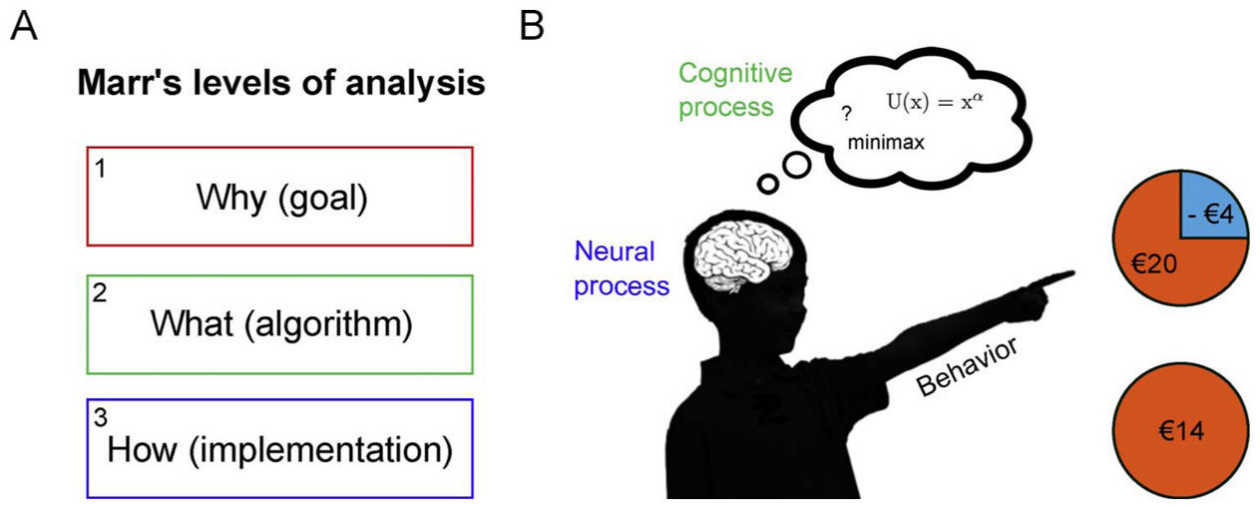
A) Marr’s levels of analysis. B) Cartoon of a child choosing between two wheels of fortune. A risky option with a 75% chance of winning 20 Euro and 25% chance of losing 4 Euro versus a safe option with 100% chance of winning 14 Euro. Choosing the option with the highest outcome variance (the risky option) is often considered risk seeking behavior in the context of these tasks (even when total expected value is the same).

David Marr’s levels of analysis approach (Fig. 1A) is probably the best-known framework devised to formalize our understanding of brain-behavior relationships (Marr and Poggio, 1976). In this approach, the first level is more abstract and is concerned with the “Why” question of behavior (e.g., why would it be beneficial for adolescents take more risks?). The “Why” question may inspire experimental design that can help understand when a particular behavior occurs. For instance, if risk taking is a form of costly signaling, showing off your strength to gain social status in the group, it should occur specifically in presence of relevant peers. Computational analyses of behavior would target Marr’s algorithmic level (the “What” question). The algorithmic level focuses on the rules that underlie behavior (e.g., risk preference can be formalized as calculating expected utility (EU) or minimax choice rules, see below for more detail). Finally, the third level (“How” question) refers to the implementation of the algorithm. What is the neurobiological substrate that supports this behavior? Marr famously argued that it is impossible to bridge the gap from the implementational level (neural processes) to behavior (risk seeking) without referring to the algorithmic level, because neural processes per se do not tell us anything about the algorithm they implement (see also (Krakauer et al., 2017)).

Following this line of argument, we argue that computational modeling can foster the understanding of lifespan development by providing testable theories that provide a bridge between Marr’s levels of analyses. One key advantage of computational models is that they allow us to capture latent variables that cannot be directly observed from behavior. As a result, by using computational models, we are better able to build specific theories about lifespan development and to identify processes that underlie developmental changes in behavior. In comparison to purely descriptive theories, the use of computational models can lead to substantially different predictions about behavior and explanations about the underlying processes. We will illustrate our arguments with specific examples in the context of decisions from descriptions (risk-taking and strategy selection) and reinforcement learning. We will also discuss several problems and potential solutions that arise when applying computational modeling to data of different age groups. Finally, we conclude with a discussion of the prospects of neuro-computational approaches in terms of our understanding of developmental changes across the human lifespan.

## 2. Models of judgment and decision-making

There has been a considerable increase in the use of paradigms from behavioral economics to study developmental changes underlying judgment and decision making. For instance, several developmental fMRI studies used risky decision making paradigms in which participants have to make repeated choices between safe and risky monetary gambles (for review see Crone et al., 2016). In most cases the aim of these studies was to investigate the developmental trajectories of risky decision making with the prediction that increased risk taking in teenagers results from an imbalance in the neural systems of motivation and cognitive control. In almost all of these studies, the analytic approach relied on objective measures of risk, e.g. comparing high versus low probability gambles or options of different expected value (EV: probability (p) multiplied by outcome (x)). However, since the development of prospect theory (Kahneman and Tversky, 1979) we know that the subjective evaluation of outcomes in most cases does not match the (objective) expected value of choice options. Furthermore, it is very likely that the subjective judgment of outcomes and probabilities differ between age groups (Eppinger et al., 2009; Harbaugh et al., 2002; Paulsen et al., 2011; van den Bos et al., 2012).

One way of addressing these issues is to refer to expected utility (EU) models, such as cumulative prospect theory (CPT; (Tversky and Kahneman, 1992)), that explicitly account for the fact that people do not behave as EV models would predict. One key notion of EU models is that of decreasing marginal sensitivity. That is, outcomes have decreasing marginal effects as more is gained or lost (see Box 1). For instance, this implies that receiving 10 Euro when you have nothing is subjectively experienced as a bigger gain than receiving 10 Euro when you already have 10.000 Euro on your bank account. This principle is very similar to the notion in psychophysics that the objective intensity of a stimulus is distinct from the subjective intensity that guides behavior (Fechner, 1966). According to most EU models the transformation of an outcome into its utility (U) is described by a power function

(1) $$U(x)=x^{\alpha }$$

where in a monetary gamble x denotes the objective amount of money, and where 0 ≪ *α* ≪ 1 denotes the degree that utility is diminished when the amounts of money increase. Thus, *U*(*x*) is a concave value function that describes the degree of risk-averse behavior. Note that because the function is concave, the subjective utility of gaining 20 Euro is only around 10 on the subjective utility scale. As a result, a risky prospect of gaining 20 Euro (at 10% chance) is not so attractive compared to a sure 2 Euro, even if objective EV is equal. CPT extends the more primitive EU value functions by adding three additional assumptions; (1) that there is a distinction between sensitivity for gains and losses, (2) that gains and losses are defined relative to a reference point, and (3) that there is asymmetry in the steepness of the value functions for losses and gains (losses loom larger than gains, see Box 1).

Box 1 **Cumulative Prospect Theory**

Cumulative Prospect Theory (CPT) suggest the following value function:

$$U(x)=\left\{ \begin{array}{cc} x^{\alpha }, & x\geq 0\\ -\lambda {\left(-x\right)}^{\beta }, & x\ll 0 \end{array}\right.$$

where in a monetary gamble x denotes the objective amount of money, and where 0 ≪ *α* ≪ 1 denotes the degree that utility is diminished when positive amounts of money increase, and where 0 ≪ *β* ≪ 1 denotes the degree that utility is diminished when the amounts of monetary losses increase. In addition, the λ parameter captures the psychological difference between losses and gains (losses tend to loom larger than gains).

Besides a subjective value function, CPT also assumes a psychological transformation of objective probabilities, p, into subjective probabilities, *π* (*p*). For simple gambles with just two possible outcomes subjective probabilities are expressed as:

$$\pi \;(p)=\frac{p^{\gamma }}{p^{\gamma }-{\left(1-p^{\gamma }\right)}^{1/\gamma }}$$

where the parameter γ indicates the strength of the transformation of probabilities. The typical shape of the weighting function suggests that people overestimate small probabilities and underestimate high probabilities. Finally, like most other utility models the expected utility EU of a gamble is the product of *U* (*x*) and *π * (*p*).

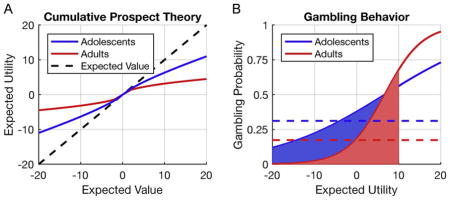

A) Fit of CPT for a set of choices by hypothetical adolescents and adults. B) Two response probability curves for two fictitious groups of adolescents and adults that only differ in their level of choice sensitivity (see Eq. (2) in text), but have the exact same EU estimations. Note that in this example the mean level of risky choices (as indicated with the dashed lines) will be higher for the adolescents than the adults.

In heuristic models of adolescent risk taking it is often assumed that an increased proportion of risky choices is the result of increased reward sensitivity. However, in many cases this relationship is only loosely defined and therefore it is difficult to make specific predictions about choice behavior or neural measures (van den Bos and Eppinger, 2016). In contrast, CPT suggests that differences in risk preferences between age groups can be attributed to different mechanisms. Let us consider a possible gamble such as the one displayed in Fig. 1B: Option A is associated with a 75% chance of winning 20 Euro and a 25% chance of losing 4 Euro. In contrast, option B represents a guaranteed win of 14 Euro.

The risky option may be more attractive to an adolescent (AD) compared to a young adult (YA) because of:

1. adolescents show higher gain sensitivity (α_AD_ ≫ > α_YA_). That is, 20 Euro are subjectively more valuable to the adolescent; one possible interpretation of “reward sensitivity”,
2. adolescents show decreased loss sensitivity (β_AD_ ≪ β_YA_). Thus, losing 4 Euro is subjectively less aversive to the adolescent,
3. reduced loss aversion (λ_AD_ ≫ > λ_YA_). That is, the psychological difference between gains and losses in adolescents is smaller compared to adults,
4. a combination of the above.

What this illustrates is that using EU models can help identifying the specific psychological mechanisms underlying developmental differences in risky choice (see also (van Duijvenvoorde et al., 2015) for a similar approach). As a result, they can also help to better understand how contextual modulations, such as peer presence (Albert et al., 2013), impacts risky choice behavior (e.g., peer presence may specifically alter loss aversion). Indeed, several behavioral studies have already identified that there are significant age differences in both probability weighting (Harbaugh et al., 2002) and value functions (Blankenstein et al., 2016; Tymula et al., 2012; van den Bos and Hertwig, 2017) between adults and adolescents.

In most cases utility models are combined with the so-called softmax choice rule that assumes that expected utilities of the options under consideration are probabilistically translated into choices. For instance, the probability of choosing a risky option, when confronted with a safe and risky alternative, would be formalized as follows:

(2) $$P_{\mathrm{risky}}=\frac{1}{1+e^{-\theta (EU_{\mathrm{risky}}-EU_{\mathrm{safe}})}}$$

where the single free parameter θ in this function governs the sensitivity to differences in EU. When participants are less sensitive to EU differences, their preferences become less consistent. For instance, let us compare the hypothetical choice functions in Box 1. The blue curve for adolescents is clearly “flatter” compared to the red curve describing the adults. This flatness indicates a decreased sensitivity to differences in EU. Both groups will prefer the option with the highest EU but adolescent will be less consistent in their choice behavior.

As a result, even when two groups may have the same ‘risk aversion’ parameter (α) but different noise parameters (θ, like the hypothetical adolescents) it is possible that one group will show a higher proportion of risky choices. This is strongly dependent on the choice set given to the participants. In Box 1, we have indicated a hypothetical choice set of gambles for which EU_safe_ − EU_risk_ = [−20, 10]. The shaded areas indicate the proportion of risky choices for each group, given that choice set, the dotted lines indicate mean levels of risk taking. If we would just compare mean levels of risk taking in this hypothetical experiment, we would conclude that adolescents are more risk-seeking than young adults. Yet, in fact, their behavioral differences are due a diminished sensitivity to outcome differences. Importantly, note that with a different choice set, where EU_safe_ − EU_risk_ = [−10, 20], the adolescents will still be closer to 50% risky choices but then will appear as more risk averse. Thus, this suggests that relying solely on choice proportions, or even just on EV models of risky choice can lead to substantial misinterpretations of age differences in risky decision making. Computational approaches using EU models can help to avoid these misconceptions, and can be used to identify the specific mechanism that contributed to changes in risk behavior.

### 2.1. Implications for imaging

The notion of EU has far reaching implications for neuroimaging studies. There is ample evidence that there are brain regions, like the ventral striatum and ventromedial prefrontal cortex, that track EU in choice experiments (Kable and Glimcher, 2007; Peters and Büchel, 2010). As we have pointed out above, EU may differ significantly from EV. Thus, if researchers use EV instead of EU as a parametric regressor in their imaging analyses of risky gambles, this will result in a better fit for those subjects for which EV and EU are most closely related (e.g., an α close to 1). Using EV will therefore be potentially misleading if age groups differ in how close their EV function is to the EU function.

For example, let’s assume that the utility curve in adolescents more closely resembles the EV of the presented decision options and, let’s assume that activity in the ventral striatum tracks the EU (rather than the EV) of options. In an analysis that only considers the EV of options the result might be greater EV-related activity in the ventral striatum in teenagers compared to adults and the conclusion would that adolescents show greater reward-sensitivity during risky decision-making. However, if we would run the same analysis with EU instead of EV we might find a very different result. Namely that there are no age differences in EU-related activity in the ventral striatum (for another example see Box 2).

Box 2 **Expected Utility and Risk Taking**

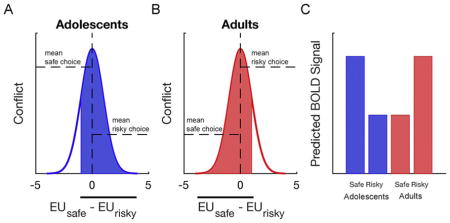

A) & B) Based on subjective utility differences the same choice set may result in mostly risky choices for adolescents and mostly safe choices for adults. Studies have shown that choices that are close to the indifference point (the point at which each option is equally preferred) are associated with increased reaction times (Krajbich et al., 2015), which is thought to reflect increased choice conflict (Botvinick, 2007). As a result, the most infrequent choices for each age group are associated with increased levels of choice conflict. C) In this example one may predict that adolescents show more BOLD activation in conflict monitoring regions (e.g., dorsal anterior cingulate cortex) when choosing the safe option compared to adults. This can falsely be interpreted that in general adolescents need more “control” to avoid risk, although it is a specific effect due to the choice set that is used. This illustrates the usefulness of thinking about our choice experiments in terms of EU. A second implication is that what on an objective level seems like the same set of questions may be very different on the subjective level. This could result in rather imbalanced choice patterns introduce various unexpected and undesirable confounds in the experimental design and the post-hoc exclusion of subjects (e.g., those who chose the safe option over 90% of the time). A simple solution is to let participants perform a pre-test to estimate parameters of the utility model, and subsequently generate unique choice sets for each individual that are equally distributed around the EU indifference point (e.g., van den Bos et al., 2015).

Of course, there are more complex risk tasks in which the probabilities and outcomes have to be learned by experience, such as the Balloon Analog Risk Task (BART) or the Iowa gambling task (IGT). It is possible that these paradigms have more predictive validity in terms of real world risk taking (Schonberg et al., 2012; van den Bos and Hertwig, 2017). However, these tasks partly depend on learning from experience and come with additional challenges in terms of computational modeling, which will be discussed below. Before turning to those issues, we will consider an alternative approach to decisions from description that focuses on the different strategies that individuals of different age might engage in when making choices

## 3. The strategy view

The expected utility models discussed above represent an integrative approach that captures individual and developmental differences using specific parameterizations within a single model. The strategy view provides an alternative framework in that differences between individuals or across development are conceptualized as the use of fundamentally different strategies. Strategies can be thought of as sequences of operations or building blocks that can be combined to solve a particular task (Mata et al., 2015). The strategy view has been used widely and successfully to understand the lifespan development of memory, arithmetic, as well as judgment and decision making (Mata et al., 2012a; Siegler, 1999).

**Fig. 2:**
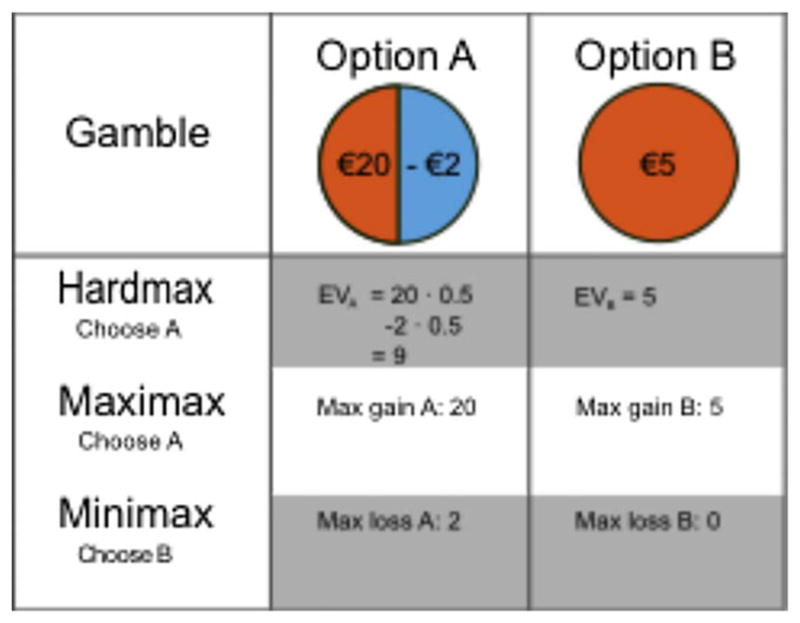
When presented with a simple binary choice gamble there are different strategies. The hardmax choice rule deterministically chooses the option with the higher expected value. Other strategies, such as minimax and maximax, only use part of the information that is presented. Maximax tries to maximize to maximum possible gain and minimax tries to minimize the maximum possible loss. Although they may yield seemingly similar behavior, they rely on different mental processes. Furthermore, even though these strategies may sometimes be captured by a parametric model such as cumulative prospect theory parameterizations (Pachur et al., 2017), they make different predictions about what happens on algorithmic and neural level (e.g., no representation of expected utility, no integration of possible outcomes).

There is a long-standing tradition in decision making research to distinguish between different strategies or heuristics (Shah and Oppenheimer, 2008). Two broad classes of strategies include compensatory strategies, that process all relevant information and consider possible trade-offs between attributes of decision options, and non-compensatory strategies, that avoid such trade-offs and typically reduce information processing demands by ignoring potentially relevant information (Gigerenzer and Gaissmaier, 2011; Payne et al., 1988). One example of a non-compensatory strategy is a lexicographic strategy that simply selects the alternative that is best on the most important attribute (e.g. the best possible outcome regardless probability, see Fig. 2). On the other hand, the previously described CPT is an example of a compensatory strategy that uses all possible information to come to a decision.

Formally, non-compensatory and compensatory strategies can be distinguished by how they assign weights to different cues in a decision problem, say deciding between two alternatives characterized by two or more cues (e.g., probability and outcome magnitude). Consider an ordered set of cues, C_1_ to C_M_. A non-compensatory strategy is defined by assigned weights to each cue, W = {w_1_, w_2_, w_3_, …, w_M_}, in a manner that each weight is larger than the sum of the subsequent weights. For example, in a five cue example, the set {1, 1/2, 1/4, 1/8, 1/16}, fulfills the requirement that for every cue weight 1 ≤*j* ≤ *M* we have *W_j_*≫ > Σ*_k_*_≫ >_*_j_ W_k_*. These weights amount to making a decision whenever the first cue distinguishes between the two alternatives and ignoring all other remaining cues, because the subsequent weights cannot “overrule” the first.

Importantly, the strategy view typically emphasizes not only the specific cue weighting but also the importance of the sequences of processes. Therefore it will typically make predictions about other outcomes beyond choice, including search (Scholz et al., 2015), reaction times (Bröder and Gaissmaier, 2007) or neural processes (von Helversen et al., 2014), that can further constrain developmental theories. These additional predictions can help to identify the underlying choice strategy that cannot be distinguished based on choice proportions or patterns alone (see also Table 1).

The empirical work that has adopted the strategy approach suggests that compensatory and non-compensatory strategies may tap into different abilities, including the ability to inhibit irrelevant information or integrate many pieces of information that show important individual differences and developmental trends across the lifespan (Betsch et al., 2016; Huizenga et al., 2007; Mata et al., 2011; Mata et al., 2010; Mata et al., 2007; Pachur et al., 2009). For example, young children seem to have difficulties using “simple” non-compensatory decision strategies due to deficits in cognitive control abilities that develop relatively late during childhood and that are necessary to inhibit accessible but irrelevant information (Mata et al., 2011). All in all, such results emphasize the importance of understanding the various strategies available to decision makers as well as the abilities that such strategies exploit which undergo substantial change during childhood and aging.

### 3.1. Implications for imaging

These age differences in learning and decision-strategies are important to take into account when analyzing neuroscience data. Knowing that different age groups may apply different strategies, and thus rely on different cognitive processes, will be extremely insightful in understanding different patterns of neural activity. This point is nicely illustrated by a recent fMRI study by van Duijvenvoorde and colleagues (van Duijvenvoorde et al., 2016). In this study participants were presented with a set of gambles designed to identify compensatory and non-compensatory strategies in risky choice. The results revealed that participants who applied a compensatory strategy showed a pattern of activity in the parietal cortex that reflected differences in EV between choice options. In contrast, for those who seemed to apply the non-compensatory strategy, their activation in the dorsomedial prefrontal cortex associated with greater conflict on the attribute level. If the authors would have used a single model to interpret the patterns of activity, it would have most likely led to a misguided interpretation.

The examples discussed above suggest that it may be helpful to distinguish different types of strategies when understanding the lifespan development of judgment and decision processes. The strategy approach, however, is not without empirical and conceptual challenges. For example, empirically, acknowledging heterogeneity in strategy use forces researchers to collect appropriately large samples to capture the cognitive and neural processes of each of the hypothesized strategy types. Conceptually, researchers face the problem of determining the space of hypothesized strategies a priori so as to avoid the problem of dealing with an intractable large number of strategies – the strategy sprawl problem (Scheibehenne et al., 2013). Fortunately, there are several possible approaches to deal with these challenges. We present an overview of the main problems, possible solutions, and some references to past exemplary work in Table 1.

## 4. Reinforcement learning models

In the previous two paragraphs, we primarily focused on decisions from description, that is, tasks in which all the information that is necessary for making the decision is available (e.g., information about the value and the probability of an outcome, see Figs. 1B and 2). In the world outside the laboratory such scenarios exist, for example, when making decisions between two different medical treatments with known risks and benefits. However, they are relatively rare; in most cases, we have to learn the expected value (EV) of choice options from experience (Hertwig and Erev, 2009). Moreover, we live in dynamically changing environments, which means that we have to constantly adjust our expectations. To do so, we often rely on trial and error learning processes, which undergo substantial developmental changes over the life course. In recent years, several research groups became interested in these processes and applied a range of experimental tasks (e.g., IGT, multi-armed bandit or reversal-learning tasks) to study different aspects of experiential learning. In most of these studies, researchers relied on descriptive summary statistics in their analyses (e.g., percentage of correct choices; (Cauffman et al., 2010; Denburg et al., 2005; Eppinger et al., 2008; Eppinger et al., 2009; Hämmerer et al., 2011; Hooper et al., 2004)). However, the use of these performance measures may result in imprecise or even misleading interpretations of the underlying computational and neural mechanisms. For example, children and older adults often show similar limitations in learning under uncertainty. However, the computational mechanisms that lead to these learning impairments may differ substantially between groups (Hämmerer and Eppinger, 2012), which may not be evident from descriptive analyses alone. To study learning under uncertainty researchers often use probabilistic choice tasks in which participants have to learn the EV of different options based on probabilistic reward (e.g., reward in 80% versus 60% of the cases). In such tasks, similar performance levels in children and older adults may either emerge from estimating the EV of the choice option too rapidly, that is, by ignoring the recent outcome history or by learning too slowly, i.e., by considering too much of the outcome history (Nassar et al., 2010). From a psychological point of view, learning too rapidly can be described as a tendency to change expectations about choice options too quickly. In the above described choice task example, this means that a participant is heavily influenced by each outcome and too eager to shift to a different choice option when the outcome does not match the expectation. In contrast, learning too slowly means that outcomes of decisions are not sufficiently considered. Thus, the learner tends to ignore the feedback. Alternatively, similar performance levels in children and older adults could be due to opposing exploration strategies. For example, consider yourself grocery shopping in a super market in a new (unknown) country. You will have to decide between various different types of e.g. cereals. Without any prior knowledge, the best thing that you can do is to sample (explore) the different options that you have. However, exploring too much results in choosing too many low-reward options, whereas exploring too little can lead to missing out on preferable options (Wilson et al., 2014). What these examples mean to illustrate is that being able to assess these different updating and exploration strategies may allow us to better understand and disambiguate lifespan age differences in learning and decision-making.

As outlined in (Hämmerer and Eppinger, 2012) it may also be that different underlying neurobiological mechanisms contribute to the seemingly similar performance profiles in children and older adults. As such, the observation that different age groups show the same performance impairments may not be sufficient to conclude that these are due to changes in similar neurobiological processes. As we argued above, computational models may help researchers to develop and test more specific theories about the mechanisms underlying developmental differences in learning and decision making and to identify the cognitive and neurobiological processes from which these differences emerge. In the following, we describe a basic computational implementation of reinforcement learning (RL), the so-called Rescorla-Wagner model and how it can be applied to study learning processes across development and aging (Daw, 2011, 2014; Niv and Schoenbaum, 2008; Sutton and Barto, 1998). RL theory offers formal models for learning from interaction with the environment when an individual has no direct instructions as to what actions to take. Accordingly, previous experiences of reward are used to form expectations about outcomes of future choices.

**Fig. 3:**
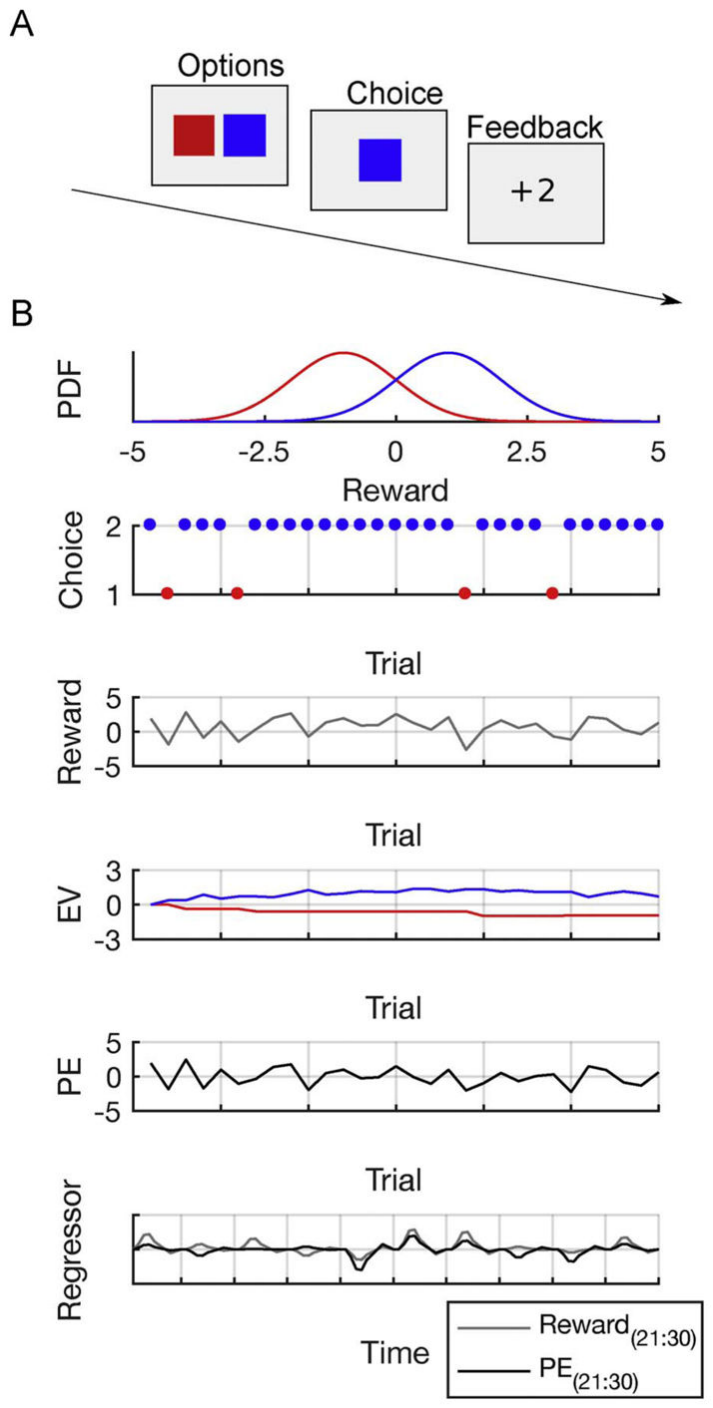
Reinforcement learning (RL) and model-based fMRI analyses. A) Example of a two-armed bandit task. Participants are required to choose between the blue and red option, which is followed by a reward or a punishment. B) Reward distribution of the blue and the red option and application of an RL model. On average, the blue option is associated with reward, the red option is associated with punishment. Choices indicate that the model is able to learn that the blue option is associated with higher reward. As a consequence of a preference for the blue option, the model receives rewards on most trials. Across trials, the model approximately learns the expected value (EV) of both options. The prediction error (PE) indicates the difference between received rewards and EVs and can be utilized to adjust EVs. Finally, model-parameters can be used as parametric regressors in neuroimaging analyses. Note that the predicted BOLD signal of rewards and PEs can go in opposite directions.

The Rescorla-Wagner model (and its many derivatives) relies on a simple principle of updating expectations based on prediction errors (Daw, 2011; Sutton and Barto, 1998). This model is often applied in simple tasks as in the example above (Fig. 3A), where participants are required to repeatedly choose one of two options (e.g., blue (B) versus red (R)) that provide probabilistic feedback, with the goal of maximizing rewards.

We assume that participants start the with a certain expectation about the two options (e.g., the EV is 0 for both B and R (Fig. 3B)) and subsequently use trial and error information to approximate the EV of each option. The basic idea of the Rescorla-Wagner model is that value expectations are sequentially updated based on the difference between the EV and the received reward: the prediction error *δ*

(3) $$\delta_{t}=r_{t}-Q_{t}(c_{t})$$

where *r_t_* is the received reward at time t and *Q_t_*(*c_t_*) is the EV of choice *c_t_* (B or R) at time *t* (see Fig. 3B). This prediction error will subsequently be used to update the EV associated with choosing option B or R

(4) $$Q_{t+1}(c_{t})=Q_{t}(c_{t})+\alpha \cdot \delta_{t}.$$

Thus, in this algorithm, the EV is updated in the direction of the prediction error to improve the accuracy of expectations. The prediction error is multiplied by a learning rate *α* to scale the influence of the prediction error on the updated stimulus value. A high learning rate (~1) will lead to an update in favor of the most recent outcomes whereas with a low learning rate (~0) the stimulus value is less affected by the outcome. Thus, the learning rate parameter specifies to what extent new outcomes affect reward expectations and can dissociate participants who learn rapidly from participants who learn slowly (see example above). In addition, in most of the learning scenarios you have two or more options that you can choose between (as in the example above). Thus, in order to make a decision you have to compare the value of the options and translate these values into choice probabilities. In many RL applications, this is achieved with the softmax function as described above (eq. 2). This parameter is often called exploration term and thus determines the degree to which the choice options are explored (see grocery shopping example above).

**Fig. 4:**
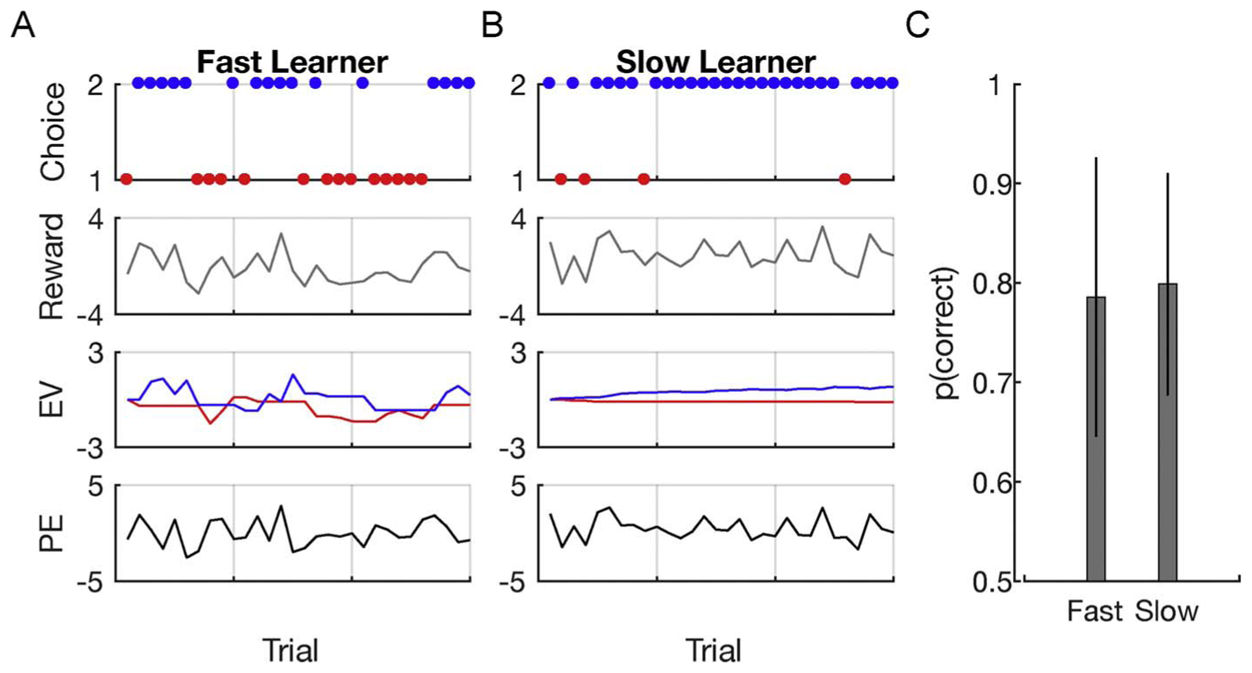
Simulations using a reinforcement learning (RL) model with different learning rate but equal exploration parameters. A) Rapidly learning RL model with a learning rate *α* = 0.6 and exploration term *θ* = 5. B) Slowly learning model with a learning rate *α* = 0.05. C) Although models have different learning rate parameters, mean performance across 500 simulations with 30 trials each is similar. Error bars represent the standard deviation of the mean between the simulations.

To illustrate how RL models can be used to dissociate behavioral performance profiles in different groups we can simulate behavior of a fast and a slow learner (a learner with a high or low learning rate): Fig. 4 shows behavior of the two RL models (Fig. 4A,B) and the average performance (Fig. 4C) across simulations in the above descried two-armed bandits task. The comparison is based on 500 simulations each consisting of 30 trials. Both models haven an exploration parameter *θ* = 5. However, behavior of model 1 (Fig. 4A) was generated using a high learning rate (*α* = 0.6) with the effect that the EV of model 1 fluctuates wildly. Model 2 (Fig. 4B), in contrast, uses a low learning rate (*α* = 0.05), which consequently leads to a slow change in the EV. As can be nicely seen, although the underlying parameters are clearly different, the average performance of both models is similar.

This simple example demonstrates both promises and pitfalls of computational modeling. It suggests that observed behavioral effects can potentially be generated by different combinations of parameters that may be captured using computational models. However, it also demonstrates the need for careful experimental design: To reliably identify parameter differences between groups or individuals, one needs to carefully think about experimental task conditions that allow to dissociate these parameters (see also Box 3).

Box 3 **Computational Modeling of Lifespan Differences**

As outlined above there are several advantages of the application of computational models in developmental cognitive neuroscience studies. However, in practice, there are also many technical hurdles that can impede these lofty research goals. In the following section, we will focus on a few of these issues that are particularly relevant for developmental comparisons.

**Model comparison**

Identifying a suitable model of behavior generally requires comparing the fit of several candidate models. For example, several researchers compared RL models with a single learning rate and models with a separate learning rate for positive and negative outcomes (van den Bos et al., 2012). Intuitively, model comparison generally involves some assessment of how likely candidate models would be to generate the observed behavior; however, another important consideration is model complexity. Models that have more freedom to explain nuances in behavior (and nuances in measurement noise) should be penalized for any performance gains achieved through overfitting (capturing variability in the noise). This is often done using penalized likelihood-based metrics such as the Akaike information criterion (AIC) and Bayesian information criterion (BIC), which penalize complexity based on parameter number and can be appropriate under a circumscribed set of assumptions. A more robust approach is to compute out-of-sample likelihoods through k-fold cross-validation or leave-one-out procedures (Friedman et al., 2001). Out-of-sample techniques are robust to non-independence of data across trials and subjects, making them ideally suited for model comparison in hierarchical models where dependencies between subjects are modeled directly (Vehtari et al., 2016). In general, out-of-sample methods are justified under a wider range of conditions and can capture more nuanced forms of complexity. In either case, best practice is to always validate the model using posterior-predictive checking to verify that the “best fitting” model is likely to generate behavioral data that look like those produced by human subjects (see below).

Note also that model comparison is another way of explicitly testing different theories against each other given the data, which is, as we argued before (van den Bos and Eppinger, 2016) very hard to do with descriptive models. As pointed out in Table 1 (identification) simulations can also be used a priori to check in advance whether an experimental setting is even capable of distinguishing between selected models (Mata et al., 2007; Mata et al., 2012b; von Helversen et al., 2010).

**Posterior-predictive checking**

One of the first steps in computational analyses is to find out whether the model that we have used fits the patterns of behavior shown by the participants. Just looking at the parameter values or goodness of fit will not reveal this; an insufficient model can provide highly misleading parameterizations of behavior (Nassar and Gold, 2013; Palminteri et al., 2016). During posterior-predictive checking (Box, 1980; Gelmanet al., 1996), we use the best fitting computational models to simulate data. These simulated data are then submitted to the same descriptive analyses that have been used to analyze the acquired (participant) data (Nassar and Frank, 2016). In some cases, this procedure might reveal that the effects observed in the simulated data closely resemble those observed in subject’s data. In other cases, the comparison may reveal a shortcoming of the model that suggests that the model architecture needs to be changed in order capture the data.

**Group differences in model fit**

A relevant concern for lifespan research is the possibility that two groups of subjects (e.g., younger and older adults) might be best described by different computational models. First, this may reflect true group differences in strategy use (which may be the result you were expecting). In this case, it will not be very meaningful to select a single model, for instance the one that fits the combined data of the groups the best, and pretend that it is the generative model for all participants. However, in some cases it could be that there exists a meaningful hybrid model that includes the features necessary to describe each group, mixed with a single parameter that governs the relative contributions of each model (Daw et al., 2011; Nassar et al., 2010). For example, recent age-comparative studies using such hybrid models showed evidence for qualitative changes in model-free and model-based learning mechanisms across the human lifespan (Decker et al., 2016; Eppinger et al., 2013b).

**Individual versus group based parameter estimation**

After model selection, the free parameters of the model of interest (such as the learning rate and exploration term) are generally estimated in order obtain trial-wise latent variables (e.g., prediction error) and to identify parameter differences between groups. On one extreme, models could be fit individually to each subject, and trial-wise latent variables could be extracted according to the model that best fits that particular subject. On the other extreme, the model could be run across data for all subjects using a set of “average” parameters, such that trial-wise latent variables are computed in the same way for each subject. Each of these approaches has its shortcomings; fitting to individual subjects leads to imprecision from limited trial data, whereas group average parameters may not provide a particularly good characterization of any particular subject and thus result in the selection of the wrong generative model (Wulff and van den Bos, 2017). A compromise between these methods is to use a hierarchical approach, such that estimates for individual subjects are constrained by their own data, as well as a group level prior that incorporates data from other subjects. In this way, parameter estimates for all subjects, but particularly those for subjects with limited or highly variable behavioral measurements, will be pulled toward the mean parameter estimate across the entire group “shrinking” the range of parameter estimates. In principle, this can be repeated at multiple levels of analysis, for example, in a nested model that includes a global prior distribution from which parameters for individual age groups are sampled. Individual parameters are then sampled from the lowest level such that they are pulled toward the mean of their own parameter distribution as well as the global mean. Note that the hierarchical approach does require making explicit modeling decisions about the prior distribution and form of hierarchy, and thus care should be taken to ensure that results are not overly conditioned on these choices (Gelman et al., 2014). That said, non-hierarchical models also make implicit assumptions about prior distributions (improper uniform) and relationships between subjects (either zero or full pooling) that could affect results.

**Linking latent computational variables to indirect neural measurements**

Once parameters are estimated, trial-wise parameters can be extracted from the properly parameterized model and linked to indirect measures of neural function such as fMRI or EEG data. This can be done by including trial-wise latent variables from the computational model as an explanatory variable in a GLM to explain trial-wise physiological measurements. One important consideration in this process is how to normalize trial-wise variables; if using either individual subject or hierarchical fitting approaches, it is important to normalize the variance (z-score) of the trial-wise latent variables for each individual subject, to ensure that detected differences in regression coefficients are not simply picking up on differences in scale emerging from the fitting process.

**Parameter recovery**

In order to interpret in differences in the best fitting parameters across groups, it is important to show that the model fitting procedures employed are capable of identifying meaningful and stable differences in any of the parameters. For instance, when there are interactions between parameters, it is possible that there are many different parameter settings that generate the same pattern of behavior. Simulating data from models that take a range of different combinations of parameter values and attempting to recover those “true” parameters by fitting the model directly to the data it generated will be informative. Only if these analyses reveal that the simulated and recovered parameter values are related in a systematic and continuous fashion they can be used for meaningful age comparisons.

**Linking computational changes to differences in behavior across developmental groups**

Once a computational model has been identified, a key question is whether the constructs captured by its free parameters (e.g. learning rate) differ systematically across groups. When data of individual subjects are fit separately (e.g., by choosing the model parameters that maximize the likelihood of the individual data), this can simply be done by comparing the group median parameter fits (e.g., using a Wilcoxon signed rank test). When interpreting a parameter difference, it is often useful to know whether behavioral differences are selective to a single computational factor (e.g., learning rate for gains but not for losses). To do so, it is important to remember that the existence of a statistical difference in one parameter and the lack of a difference in another parameter does not necessarily imply selectivity; claims of specificity should rely on explicit comparisons of age differences across the different parameters (Nieuwenhuiset al., 2011). In a hierarchical model, parameter differences between groups can be estimated by examining the posterior distribution over group differences directly, or by computing credible intervals over likely parameter differences.

Taken together, one of the advantages of computational RL approaches for developmental science is that they allow us to get access to latent variables (such as learning rates or exploration parameters) that cannot be accessed with descriptive approaches alone. These variables may help us to disambiguate behavioral profiles of different age groups and may provide us with a better mechanistic understanding of developmental differences.

### 4.1. Implications for imaging

Beyond global parameters such as learning rate or exploration parameter, which are estimated on the individual subject level, RL models can also be used to derive trial-by-trial estimates of two other latent variables: the reward prediction error *δ_t_* and the EV *Q_t_*_+1_ of the choice options (actions). When performance differences in learning occur between two different age groups, an obvious question of computational interest is whether these differences are associated with changes in reward prediction error signaling or the representation of EV in different cortical or subcortical areas. In order to answer this question, it is necessary to construct an fMRI design matrix that models the task events (e.g., decision, outcome) and includes a term that reflects the extent to which an event response is modulated by the parametric variable (the prediction error or EV, see Fig. 3B). Such a design matrix can be fit to the fMRI data using standard analysis software and the resulting coefficients for these modulator terms provide a quantitative measure of the relationship between the BOLD signal and the latent variable of interest (e.g., reward prediction error). Thus, in principle this measure can be used to test for differences in neural computations underlying learning (Fig. 3B).

Several recent studies have taken advantage of these approaches and show evidence for differences in the correlation between model-derived prediction errors and BOLD activity in the ventral striatum in children and adolescents (Christakou et al., 2013; Hauser et al., 2015; Javadi et al., 2014; van den Bos et al., 2012) as well as older adults (Chowdhury et al., 2013; Eppinger et al., 2013a; Samanez-Larkin et al., 2014). Most of this work has focused on model-free RL, using for example the Rescorla-Wagner model described above. Recently, however, there is an increasing interest in more complex types of RL (“model-based” learning), which involve learning of a forward model of the environment that can be used for planning (Daw et al., 2005; Dolan and Dayan, 2013). Model-based learning may be advantageous, especially in complex environments, because it allows more sophisticated behavioral strategies than model-free learning. However, this advantage comes at the cost of higher demands on, for example, working memory and attention, which makes it an interesting target for research in lifespan cognitive neuroscience (Decker et al., 2016; Eppinger et al., 2013b).

In a recent age-comparative study, Nassar et al. (2016) used RL principles in combination with Bayesian methods to model learning dynamics in uncertain and changing environments. Using a predictive inference task, the authors examined age-related changes in the factors that affect trial-to-trial adjustments of learning rates. The results suggested that age-related learning deficits in older adults are due to a specific deficit in representing uncertainty. This deficit may not directly affect prediction error signaling but rather the computation of the learning rate, which, as described above, determines the degree to which prediction errors are considered during learning.

To conclude, RL offers a theoretical framework to study learning and decision making across the lifespan. The key advantage of these computational models is that they allow us to estimate latent variables such as prediction errors or the learning rate that cannot be assessed using descriptive models. Another important advantage is that RL models can be used to simulate the impact of developmental differences on behavior (see Nassar et al., 2016; Palminteri et al., 2016). This leads to a considerable increase in the specificity of predictions regarding developmental differences in learning and decision processes. Finally, model parameters can be used to inform fMRI or EEG analyses which may allow to identify the neurobiological mechanisms underlying developmental changes in learning. However, despite all these potential advantages, in practice, there are also many technical hurdles that have to be tackled. In Box 3 we focus on a few of the issues that are particularly relevant for developmental comparisons.

## 5. General discussion

In this paper, we have provided an overview about how computational models of cognition can be used to study age-related changes in psychological processes and the underlying neurobiological mechanisms across the human lifespan. The core idea of this neuro-computational approach is that formalized models can provide us with mechanistic links between verbal descriptions of behavior and its cognitive and neurobiological implementations.

Specifically, we propose that computational models allow us to address two current problems in developmental cognitive neuroscience: 1) The problem of making predictions about behavior and the underlying neurobiology that are specific enough to falsify verbal theories (specificity problem) and 2) The problem of capturing the identity of developmental processes (identity problem). We have shown applications of computational neuroscience approaches in two major domains of decision making: decisions from description and decisions from experience. Here we have focused on the use of models to describe that behavior, note that there is already a wealth of developmental neuroscience studies on these topics (Hartley and Somerville, 2015).

In both of these domains computational approaches in combination with neuroimaging can significantly advance our mechanistic understanding of the underlying processes. This has led to the development of new fields such as computational psychiatry which aims to provide a mechanistic understanding of psychiatric disorders that can guide theory-based clinical interventions (Ahn and Busemeyer, 2016; Huys et al., 2016). Many of the disorders under study (such as schizophrenia, anxiety, addiction or ADHD) develop during late childhood and adolescence. Therefore, any type of complete theory utilized to explain these disorders has to incorporate a normative perspective of human development. The same is true for aging-related diseases such as Parkinson’s disease or dementia. Yet, we are far away from such neuro-computational theories of development and aging. The aim of this work is to provide a starting point for the development of such theories and to encourage researchers to adopt neuro-computational approaches.

It is obvious that there are important questions and research strategies that we have not covered. For example, there is an emerging literature on developmental differences in perceptual decision making and the use of drift diffusion models to discern different perceptual decision making processes (Ratcliff et al., 2006; Schuch and Konrad, 2017; Spaniol et al., 2006; Thompson et al., 2016). Furthermore, there is an increasing interest in studying how the effort that is involved in making a decision affects choice behavior. So far there are only a few studies on age differences in effortful decision making (Benozio and Diesendruck, 2015; Westbrook et al., 2013) and the existing computational approaches (Kool et al., 2016; Shenhav et al., 2013) have not (yet) been implemented. We have not addressed a core dilemma in decision making, the question how we solve the trade-off between exploration and exploitation and how this changes across development (Somerville et al., 2017; Wilson et al., 2014). Finally, we also ignored the extensive literature on neural network modeling of development (Mareschal and Shultz, 1996; Plunkett et al., 1997). These models provide (even) more complex perspectives on cognitive development and aging and may be well suited to address the identity problem.

With respect to the neuroscience approaches we focused on fMRI, but there are new ways of analyzing EEG data using single trial approaches which are promising (Fischer and Ullsperger, 2013) and there are several other recent examples of extremely fruitful combinations of computational modeling and psychophysiological measures (Cavanagh et al., 2014; Nassar et al., 2012).

As outlined there are several potential pitfalls when applying computational models to behavioral data and in using the outcomes of modeling to inform neuroscience data. Some of these pitfalls are specific to developmental research questions, others are more general and our review of these issues is certainly not exhaustive (for more detailed descriptions please refer to (Daw, 2011; Nassar and Frank, 2016; Palminteri et al., 2016) and to (Redish and Gordon, 2016) for an overview of uses in psychiatry). Given the increasing interest in computational neuroscience methods it is important to note that there are several non-trivial inferential problems regarding 1) whether and to which degree a model actually fits the data and 2) what correlations between computational parameters and neurobiological signals actually reflect. The latter point refers to the fact that with the current neuroimaging approaches we cannot make causal inferences about model parameters and neurobiological signals. That is, even though it may be tempting to assume that, for example, learning deficits in older adults are due to diminished striatal prediction error signals, the relationship still remains a correlative one and typically activity in several other areas also correlates with prediction errors (Hayden et al., 2011; Schultz and Dickinson, 2000). To tackle the question of causality, we will have to rely on non-invasive brain stimulation methods (e.g., rTMS) or pharmacological manipulations (Chowdhury et al., 2013), and engage in cross-species comparisons (e.g., involving optogenetic manipulations in rodents). In addition, the correlation (or lack thereof) between computational parameters and neuroscience data can also be used in model selection itself. Often the computational model is fit on the behavioral data alone, but to the extent that the model makes predictions about neural processes, model selection can also be (simultaneously) constrained by neural data. This is an exciting future direction in computational neuroscience that can further our confidence in the identification of cognitive processes underlying life span changes in behavior (Turner et al., 2013).

We are aware of the valid concern that computational neuroscience approaches may lead to a segmentation of behavior into “molecular” psychological and neurobiological processes that are, at some point, far removed from the behavior that was originally set out to be explained. That is, the jargon associated with increasingly complex computational models may become meaningless if the models cannot speak to existing psychological theories of behavior. It is therefore crucial to use our increasing understanding of the neuro-computational mechanisms to answer the question of why humans of different ages behave in a certain way, by finding ways of linking the algorithmic level to back the level of verbal theories.

Finally, it is important to point out that computational modeling cannot replace good experimental design, but rather can inform it. That is, the value of a computational model is constrained by the value of the experimental design that it is associated with. Even if the model could perfectly explain the behavior on a specific task, and the accompanying neural processes, it will be of limited value if this task has no external validity. For instance, adolescents probably only rarely encounter “risky” choices where they are presented with full information about probabilities and outcomes such as is often done in monetary gambles (see Fig. 1B). Thus, one may wonder how informative age differences in parameters of pure risky choice models will be (cf. (van den Bos and Hertwig, 2017)). In addition, experimental tasks are often simplified in order to capture one specific feature of the real-world environment or one isolated psychological process. This strategy may limit external validity because it may miss out on crucial complexities that explain real world behavior. Having a good computational model will provide a framework for understanding how multiple variables interact (and change over time) and therefore may allow for the design of more complex, externally valid experiments.

To conclude, we think that neuro-computational approaches have a tremendous potential for studying human development across the lifespan. Computational methods can provide access to latent processes that are not accessible with descriptive methods and may thus foster the development of mechanistic theories of normative development. This allows specific predictions about brain behavior relationships in different developmental groups (specificity problem) and may enable us to identify the nature of developmental processes (identity problem).

## Figures and Tables

**Table 1:** Overview of typical problems and solutions when applying the strategy view.

Problem	Possible Solution(s)
**Strategy sprawl**: There is a potentially large number of combination of core processes or building blocks that can be combined which may lead to hypothesizing an intractably large number of strategies	Use a pre-defined and constrained set of strategies that have been validated in past research (Mata et al., 2007)
	Validate the use of several strategies using additional process data, such as search (Scholz et al., 2015), reaction time (Bröder and Gaissmaier, 2007), or neural data (Fechner et al., 2016; Khader et al., 2011; von Helversen et al., 2014)
	Adopt Bayesian modeling to help quantify the trade-off between flexibility in the number of hypothesized strategies and descriptive adequacy (Scheibehenne et al., 2013)
	Explicitly model the mixture of strategies, and use parameter estimation to establish the contributions of individual strategies (Bays et al., 2009; Collins and Frank, 2012; van den Berg et al., 2014).
**Identification problem**: Different strategies may often provide the same judgment or choice	Design experimental paradigms so as to include critical tests that allow maximizing differences between hypothesized strategies (Mata et al., 2007; Mata et al., 2012b; von Helversen et al., 2010)
**Individual heterogeneity**: Heterogeneity in strategy selection across individuals may lead to few individuals using the same strategy in a given situation	Calculate power a priori based on hypothesized strategies and collect appropriately large samples
	Collapse across individuals in a meaningful way, for example, across similar strategy types, such as compensatory and non-compensatory strategies (Mata et al., 2010) or rule-based and similarity-based processes
	Design the task to elicit specific strategies (Juslin et al., 2003)
	Instruct or train individuals to execute specific strategies (Mata et al., 2009; Siegler and Lemaire, 1997)
**Ecological rationality**: Different statistical environments (e.g., cue-criterion correlations) favor different strategies and individuals adapt their strategy use accordingly, potentially masking developmental effects	Design the statistical structure of the task to elicit specific strategies (Mata et al., 2007)
	Compare different statistical structures and assess individual or developmental differences in adaptivity (Horn et al., 2016)
**Strategy execution**: Differences in judgment or choice outcomes may be due to differences in both strategy selection and strategy execution	Adopt computational models that estimate strategy execution errors (Mata et al., 2010; Mata et al., 2011)
	Estimate developmental effects on strategy selection and execution directly using choice vs. no-choice method, that is, comparing experimental conditions in which individuals can select (choice) or simply execute (no-choice) particular strategies (Siegler and Lemaire, 1997)
